# Total marrow irradiation (TMI): Addressing an unmet need in hematopoietic cell transplantation - a single institution experience review

**DOI:** 10.3389/fonc.2022.1003908

**Published:** 2022-10-03

**Authors:** Jeffrey Y.C. Wong, An Liu, Chunhui Han, Savita Dandapani, Timothy Schultheiss, Joycelynne Palmer, Dongyun Yang, George Somlo, Amandeep Salhotra, Susanta Hui, Monzr M. Al Malki, Joseph Rosenthal, Anthony Stein

**Affiliations:** ^1^ Departments of Radiation Oncology, City of Hope, Duarte, CA, United States; ^2^ Department Computational and Quantitative Medicine, City of Hope, Duarte, CA, United States; ^3^ Department of Hematology and Hematopoietic Cell Transplantation, City of Hope, Duarte, CA, United States; ^4^ Department of Pediatrics, City of Hope, Duarte, CA, United States

**Keywords:** total marrow irradiation, total marrow and lymphoid irradiation, tomotherapy, VMAT, acute leukemia, hematopoietic stem cell transplant, bone marrow transplantation, total body irradiation

## Abstract

**Purpose:**

TMI utilizes IMRT to deliver organ sparing targeted radiotherapy in patients undergoing hematopoietic cell transplantation (HCT). TMI addresses an unmet need, specifically patients with refractory or relapsed (R/R) hematologic malignancies who have poor outcomes with standard HCT regimens and where attempts to improve outcomes by adding or dose escalating TBI are not possible due to increased toxicities. Over 500 patients have received TMI at this center. This review summarizes this experience including planning and delivery, clinical results, and future directions.

**Methods:**

Patients were treated on prospective allogeneic HCT trials using helical tomographic or VMAT IMRT delivery. Target structures included the bone/marrow only (TMI), or the addition of lymph nodes, and spleen (total marrow and lymphoid irradiation, TMLI). Total dose ranged from 12 to 20 Gy at 1.5-2.0 Gy fractions twice daily.

**Results:**

Trials demonstrate engraftment in all patients and a low incidence of radiation related toxicities and extramedullary relapses. In R/R acute leukemia TMLI 20 Gy, etoposide, and cyclophosphamide (Cy) results in a 1-year non-relapse mortality (NRM) rate of 6% and 2-year overall survival (OS) of 48%; TMLI 12 Gy added to fludarabine (flu) and melphalan (mel) in older patients (≥ 60 years old) results in a NRM rate of 33% comparable to flu/mel alone, and 5-year OS of 42%; and TMLI 20 Gy/flu/Cy and post-transplant Cy (PTCy) in haplo-identical HCT results in a 2-year NRM rate of 13% and 1-year OS of 83%. In AML in complete remission, TMLI 20 Gy and PTCy results in 2-year NRM, OS, and GVHD free/relapse-free survival (GRFS) rates of 0%, 86·7%, and 59.3%, respectively.

**Conclusion:**

TMI/TMLI shows significant promise, low NRM rates, the ability to offer myeloablative radiation containing regimens to older patients, the ability to dose escalate, and response and survival rates that compare favorably to published results. Collaboration between radiation oncology and hematology is key to successful implementation. TMI/TMLI represents a paradigm shift from TBI towards novel strategies to integrate a safer and more effective target-specific radiation therapy into HCT conditioning beyond what is possible with TBI and will help expand and redefine the role of radiotherapy in HCT.

## Background and rationale

Total marrow irradiation (TMI) and total marrow and lymphoid irradiation (TMLI) are methods to deliver organ sparing targeted radiotherapy using intensity modulated radiation therapy (IMRT). This approach offers radiation oncologists, hematologists and the bone marrow transplant team the ability to reduce dose to critical organs or any other anatomic region, while increasing dose to user-defined targets depending on the clinical situation. TMI and TMLI represent a departure from total body irradiation (TBI) and a paradigm shift in the use of radiotherapy as part of the conditioning regimen in hematopoietic cell transplantation (HCT).

The concept of TMI was first proposed in 2001. To implement this concept required close collaboration between radiation oncology and hematology, the development of treatment planning methods (2002) (1–3), the design of three initial pilot and phase 1 clinical trials (2003–2004), and culminated in treatment of the first patient in June, 2005 (4, 5) using a TomoTherapy HiArt System®. TMI and TMLI were originally developed by our group to address an unmet need in patients undergoing HCT, specifically patients with advanced, refractory or relapsed (R/R) hematologic malignancies who have poor outcomes with standard conditioning regimens, who could not tolerate standard total body irradiation (TBI), and where attempts to improve outcomes by adding or dose escalating conventional TBI was not possible due to associated toxicities. Clinical settings where TMI and TMLI were felt to have potential application included 1) patients older than 60 years of age who cannot tolerate standard TBI; 2) patients who have poor outcomes with reduced intensity conditioning (RIC) regimens and where the addition of TBI to RIC is not feasible (6); and 3) patients with R/R acute leukemia where dose escalation of TBI reduced relapse rates but also increased toxicities, negating any gains in survival (7). The emphasis was on redefining and expanding the use of radiation containing conditioning regimens to a new group of patients, in addition to reducing toxicities from conventional TBI for existing patients.

Since the first patient was treated with TMI in 2005, over 500 patients have been treated at this center. This review will summarize this clinical experience; the total doses, dose distributions, and fractionation schedules used; the approach to planning and delivery; the rationale and results of past and current trials; and perspectives on the future directions in this field.

## Implementation of TMI

### Initial treatment planning studies

The development of TMI at this center from initial concept to treatment of the first patient spanned 4 years and involved from the beginning collaboration between radiation oncologists, medical physicists and hematologists. We proposed the concept of TMI to TomoTherapy, Inc. in 2001, prior to FDA approval of the TomoTherapy Hi-Art system in 2002. Initially, the TomoTherapy Hi-Art system was not designed to plan and deliver TMI. Software and hardware modifications to the treatment planning system had to be made by the manufacturer since TMI planning required large complex data sets due to the need for whole body imaging and multiple organ contouring on a scale that had not been attempted before.

**Figure 1:**
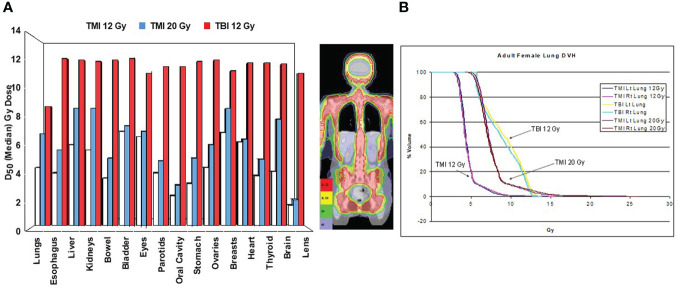
First TMI treatment plan comparison at City of Hope comparing in the same patient TBI 12 Gy, TMI 12 Gy and TLI 20 Gy (4). **(A)** Median organ doses. **(B)** Dose volume histogram curves for lung.

The first TMI treatment plan, where the target region was defined as the skeletal bone, was based on a whole-body CT data set of a 20-year-old woman with acute myelogenous leukemia (**Figure 1**). Treatment planning studies compared TMI to standard TBI using 50% transmission lung blocks with electron boost to the underlying chest wall. This demonstrated median organ doses with TMI that were 20-70% of the target dose, significantly less than TBI where most organs received 11.5-13.1 Gy and the shielded lungs 9.0-9.7 Gy. This predicted for a reduction of acute and late toxicities compared to TBI. At TMI doses up to 20 Gy, median doses to all organs were still below that of TBI to 12 Gy (**Figure 1A**). At 20 Gy TMI lung dose volume histogram (DVH) plots (**Figure 1B**) demonstrated the minimum dose to at least 80% of the lung volume (D_80_) was comparable to 12 Gy TBI.

These initial plan comparisons determined radiation dose distributions and organ dose constraints for the initial clinical trials. These plan comparison studies also helped to communicate the concept and advantages of TMI and TMLI to our hematologists, leading to clinical trial development, acquisition of the first TomoTherapy system and the treatment of the first patient. The highest prescribed dose level on phase I trials was limited to 20 Gy since lung doses began to approach that of conventional TBI with lung shielding (4), predicting for comparable risks of pneumonitis (8). For all trials the mandible was not included as a target structure to reduce the likelihood of severe oral mucositis, which was a primary toxicity of the myeloablative TBI and etoposide regimens used at this center. TMLI preceded chemotherapy as with standard TBI regimens used at this center. Fraction size and schedule was 1.5 to 2.0 Gy twice a day with a minimum of 6 hours between fractions, a fractionation schedule widely used to deliver TBI.

**Figure 2:**
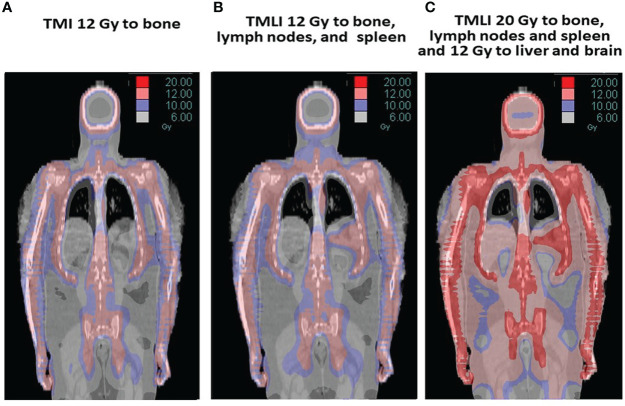
The term TMI is used if the target is bone **(A)** and was used in a tandem autologous HCT multiple myeloma trial (9, 10). TMLI adds the major lymph node chains and spleen as target regions and is used in allogeneic HCT regimens **(B)** (11). In some studies, TMLI also includes the liver and brain to 12 Gy while other target regions (bone, lymph nodes and spleen) are escalated to 20 Gy **(C)** (12, 13).

**Figure 2** displays radiation dose distribution patterns used at this center. The term TMI is used if the target structure is bone and was used in an initial tandem autologous HCT trial in patients with advanced multiple myeloma (**Figure 2A**) (9, 14). The term TMLI is used when the major lymph node chains and spleen are added as target regions to achieve the immunosuppression needed for allogeneic HCT and is used at this center primarily for patients 60 years of age or older (**Figure 2B**) (11, 15). In patients younger than age 60 undergoing allogeneic HCT, liver and brain are added as target regions to TMLI (**Figure 2C**) (16).

TMI and TMLI have been delivered on clinical trials at this institution so that data could be prospectively collected to address questions and concerns prior to treating the first patient. Was the delivery of TMI and TMLI feasible and safe? Would the higher dose rates compared to conventional TBI increase organ toxicities, graft versus host disease (GVHD), or engraftment failure? Would dose reduction to organs at risk (OARs) and the helical tomographic or volumetric modulated arc radiotherapy (VMAT) delivery of radiation therapy spare circulating leukemia cells and lead to increased relapse rates?

### Simulation, planning, and treatment delivery

CT simulation is performed in the supine position. Immobilization is with a body vac-lok™ bag (CIVCO Medical Systems, Kalona, IA) from the base of neck to the feet, a type-S thermoplastic head frame (CIVCO, Kalona, Iowa, IA), and Accuform™ cushion to immobilize the head and shoulders. CT scans are obtained using 5-8mm slice thickness. A body CT scan is obtained with normal quiet breathing and is used of treatment planning. 4D CT scans of the chest and abdomen are utilized to account for any organ motion with respiration. Radiopaque markers are placed at mid-thigh to identify the junction for planning. If treatment will be on a Tomotherapy unit, the patient is positioned with the top of the head 5 cm from the end of the couch and the couch height is 10 cm below the isocenter of the gantry.

Contouring is done on an Eclipse workstation (Varian Medical Systems, Palo Alto, California). The following organs are contoured in all patients: lungs, heart, kidneys, liver, esophagus, oral cavity, parotid glands, thyroid gland, eyes, lens, optic chiasm and nerves, brain, stomach, small and large intestine, breasts, rectum, testes, ovary and bladder. Target structures are defined by the treatment team and clinical trial. The 4D CT datasets are used for contouring the ribs, kidneys, spleen and liver to account for any respiratory motion. Maxillary and mandibular bones are excluded as targets to reduce oral cavity dose. A 5-10 mm margin around the CTV is usually used to define the PTV. The use of whole-body auto-segmentation software has significantly reduced the time needed for contouring.

The majority of patients to date have been treated on a TomoTherapy system. With TomoTherapy planning system optimum results are achieved when organ dose reduction is performed first followed by target dose optimization. For most plans the target receives a minimum of 85% of the prescribed dose. Most patients are treated using a jaw setting of 5 cm, modulation factor of 2.5, and pitch of 0.287. Legs and feet are planned in Tomo-Direct mode or with conventional AP/PA fields. Volumetric imaging is used for daily patient setup. On TomoTherapy, a megavoltage CT (MVCT) or kilovoltage CT (kVCT) scan is performed from the skull to iliac crest.

TMLI has also been delivered at this center using a Varian TrueBeam linear accelerator (Varian Medical Systems, Palo Alto, California) with VMAT capability (17). Four to five isocenters are used for the upper body TMLI treatment plan, with two arc fields typically placed for each isocenter. A 120-leaf multi-leaf collimator is used. The leaf width is 5 mm for the central 40 leaf pairs and 1 cm for the peripheral 20 leaf pairs. The collimator angle is at 90°C so that the MLC leaves move along the longitudinal direction of the patient. Asymmetric jaws are used along the patient’s longitudinal direction so that the two arc fields at each isocenter cover different patient body lengths. A 6-MV photon beam is used for all the VMAT fields. The lower extremities are planned and treated with junctioned AP-PA fields. For setup on the Varian TrueBeam linear accelerator, two cone-beam CT (CBCT) scans are performed, one in the head & neck region and the other in the abdominal and pelvic region.

A review of organ doses on over 200 patients treated at this center with TMLI has recently been published by Han et al. (18). **Tables 1**, **2** provide select mean organ doses for patients treated at 12 and 20 Gy TMLI. Average dose-rates are 130-180 cGy/minute to the target and 70-90 cGy/minute to the lung. TMI and conventional TBI planning and delivery at this center require similar time and resources (19). Details regarding simulation, immobilization, planning, and treatment delivery have previously been published by Liu et al. (19), Han et al. (18) and Schultheiss et al. (1, 2, 5).

**Table 1:** Summary of mean organ dose (Gy) for patients treated with 12 Gy TMLI (n = 108).

Organ at Risk	Mean Dose ± SD (Gy)12 Gy TMLI	Range (Gy)12 Gy TMLI
**Bladder**	7.60 ± 1.53	3.75 - 11.47
**Brain**	6.68 ± 0.88	5.09 - 8.99
**Esophagus**	4.95 ± 0.92	3.19 – 7.72
**Heart**	6.12 ± 1.01	3.80 – 8.39
**GI - Lower**	5.91 ± 0.87	4.61 – 9.00
**GI - Upper**	5.22 ± 0.84	3.78 - 7.82
**Kidneys**	5.68 ± 1.37	3.10 – 8.34
**Lens**	2.17 ± 0.94	1.00 – 6.93
**Liver**	7.22 ± 0.94	3.17 – 9.07
**Lungs**	6.20 ± 0.61	4.40 – 7.41
**Oral Cavity**	3.20 ± 0.61	1.78. – 5.05
**Parotids**	5.43 ± 1.12	3.41 – 9.61
**Rectum**	4.87 ± 1.00	2.36 – 7.54
**Thyroid**	5.98 ± 1.70	3.08 - 12.15

Target structures are bone, major lymph node chains and spleen (**Figure 2B**) (18).

**Table 2:** Summary of mean organ dose (Gy) for patients treated with 20 Gy TMLI (n = 120).

Organ at Risk	Mean Dose ± SD (Gy)20 Gy TMLI	Range (Gy)20 Gy TMLI
**Bladder**	9.71 ± 1.49	5.96 – 14.56
**Esophagus**	6.50 ± 0.87	3.17 – 8.73
**Heart**	7.38 ± 0.56	5.78 – 8.55
**GI - Lower**	10.15 ± 1.11	6.60 – 13.23
**GI - Upper**	9.01 ± 1.34	6.24 – 12.59
**Kidneys**	7.28 ± 0.69	5.39 – 9.08
**Lens**	2.55 ± 0.41	1.78 – 4.36
**Lungs**	8.48 ± 0.83	6.22 – 10.19
**Oral Cavity**	4.43 ± 0.97	2.95 – 7.08
**Parotids**	8.05 ± 1.31	5.64 – 12.21
**Rectum**	6.46 ± 0.88	4.58 – 9.06
**Thyroid**	8.01 ± 2.13	3.67 – 17.12

Target structures are bone, major lymph node chains and spleen with liver and brain limited to 12 Gy (**Figure 2C**) (18).

## Review of clinical results

### Initial demonstration of feasibility, acceptable toxicities and dose escalation using TMI

Somlo et al. first reported the results of a Phase I/II trials using TMI in patients with multiple myeloma undergoing autologous tandem HCT (9). High dose melphalan conditioning is often used (20, 21). Myeloablative TBI added to high dose melphalan was not feasible due to dose-limiting mucositis (22). Since this trial was the first-in-human use of TMI and given the concerns of increased toxicities due to higher dose-rate, TMI was delivered without concurrent chemotherapy and a fractionation schedule similar to standard TBI was used.

Patients with Salmon-Durie stage I-III multiple myeloma and with stable or responding disease after first line therapy were entered. Patients first received melphalan (200 mg/m^2^) followed by autologous HCT. Six to ten weeks later they received TMI to bone (**Figure 2A**) followed by a second autologous HCT. TMI dose was escalated from 10 to 18 Gy. The fractionation was 2 Gy delivered twice a day with 6 hours between fractions. Median organ doses were 11% to 81% of the prescribed bone dose (9), A maximum tolerated dose (MTD) of 16 Gy was defined based on toxicities seen at 18 Gy (1 patient with reversible grade 3 pneumonitis and 1 patient with grade 3 hypotension attributed to engraftment syndrome). This was followed by an expansion of patients treated at 16 Gy TMI (10). Of the 54 patients on this study, 30 patients (55.6%) underwent TMI at the MTD of 16 Gy. Median follow-up of surviving patients was 12.3 years (9.2 - 15.5+). Overall survival (OS) at 10 years were 38.8% with a progression-free survival (PFS) plateau of 20.4%. The first patient with stage I disease remains in remission 18 years after TMI. TMI for multiple myeloma before autologous HCT warrants further study.

### Dose escalation of TMLI in younger patients (< 60 Years Old) with relapsed/refractory acute leukemia

There is a dose response for acute leukemia. For chloromas local control rates are approximately 20% after doses < 10 Gy, 40% after 10-20 Gy and over 80% after doses > 20 Gy (23). A decrease in relapse rate with higher TBI doses has been observed (24–26). Randomized phase II single institution trials have compared 12 Gy versus 15.75 Gy TBI combined with cyclophosphamide (Cy) (7, 27). In patients with AML in first remission, TBI to 15.75 Gy resulted in a reduction in relapse rate (14% versus 39% p = 0.06), but an increase in NRM rate (38% versus 19%, p = 0.05), resulting in no difference in overall survival between the two arms (7). A higher incidence of grade 3-4 hepatotoxicity, grade 3-4 renal toxicity, grade 2 mucositis and GVHD was observed with the higher TBI dose (28, 29). These data suggest that a more targeted form of radiotherapy such as TMLI, which reduces dose to the liver, kidneys, lungs, oral cavity, esophagus and GI tract, is needed before clinically important dose escalation becomes feasible with acceptable toxicities. In addition, escalation of conditioning regimens by adding chemotherapy to myeloablative regimens in patients with active leukemia resulted in long term cure in up to 30-40% cases, indicating that escalation of conditioning intensity may induce cure in some patients with advanced leukemia (30).

Dose escalation trials at this center were initiated in patients with R/R AML and ALL. Long term OS and PFS rates in these patients are less than 20% after standard allogeneic HCT (31). TMLI, busulfan [days -12 to -8 (800 uM min)] and etoposide [day -3 (30 mg/kg)] conditioning was evaluated in a phase I trial (32). TMLI dose was 12 Gy (n=18) and 13.5 Gy (n=2) at 1.5 Gy twice daily. The target structures were bone and bone marrow, major lymph node regions and spleen. Liver and brain received 12 Gy. (**Figure 2C**). Twenty patients were treated, with 19 patients still with detectable blasts in marrow and 13 detectable circulating blasts prior to HCT. Grade 4 dose limiting toxicities of stomatitis and sinusoidal obstructive syndrome (SOS) were seen at 13.5 Gy. Hepatotoxicity was likely due the combination of busulfan and a liver dose of 12 Gy, each of which has been associated with a risk of SOS. TMLI dose escalation was not feasible with this regimen.

Stein et al. (16) reported results of a phase I trial using a conditioning regimen of escalating doses of TMLI [(range: 12-20 Gy, in 2 Gy increments) 1.2 - 2 Gy twice a day, from day -10 to day -6] with Cy (100 mg/kg day -3) and VP-16 (60 mg/kg day -5). in a phase I trial of 51 patients with R/R AML (n=33) and ALL (n=16) (NCT02446964). The target structures were bone and bone marrow, major lymph node regions and spleen. Liver and brain were kept at 12 Gy. (**Figure 2C**). Fifty patients had detectable blasts in marrow (median 52%, range 5-98% involvement) and 27 patients had circulating blasts in the week prior to HCT conditioning. Dose-limiting toxicity (DLT) (Bearman scale grade 3 mucositis) (28) was observed in 1 patient at the 15 Gy dose level. No further DLTs were observed up to 20 Gy. All patients engrafted without delays. Median follow-up was 24.6 months in surviving patients. The 1-year OS was 55.5% and PFS was 40.0%. NRM rates were 3.9% at day 100 and 8.1% at 1-year.

A subsequent phase II trial evaluating the same regimen is currently ongoing at this center (NCT 02094794). A recent analysis of the first 57 patients with AML (n=43) and ALL (n=14) treated with TMLI doses of 20 Gy reported a 1-year NRM rate of 6% and 2-year OS and PFS of 48% and 33%, respectively, which compare favorably to published results (12, 33). All patients engrafted. Mean organ doses were 20-51% of the target dose. Mean organ doses (Gy) were lung 9.1, kidneys 7.3, GI tract 10.3, esophagus 6.7, and oral cavity 4.3. In summary TMLI to 20 Gy can be safely delivered with etoposide and cyclophosphamide with low NRM rates of <10% and with encouraging PFS and OS in patients < 60 years old with R/R acute leukemia.

These trials are in patients with relapsed and refractory acute leukemia with detectable blasts in marrow and circulation just prior to TMLI. These patients have a dismal outcome after HCT with a long-term survival of 16% to 19% (31). Patients with acute leukemia who relapse after first remission are usually unable to achieve a second remission with salvage chemotherapy and have very few therapeutic options outside of clinical trials (34). Patients with relapsed and refractory acute leukemia are not transplanted at most center. Therefore, there are no consensus standard of care HCT regimens that the results of these trials can be compared to. Case examples that follow illustrate the type of patients entered on these studies.

*Case example. A 29-year-old female with relapsed AML in marrow, CNS and lymph nodes. The patient initially achieved complete remission after 7 + 3 induction and 1 cycle consolidation with high dose cytarabine. She developed right orbital and CNS relapse. She received intrathecal chemotherapy followed by whole brain radiotherapy to 12 Gy. She then developed back and left leg pain from enlarged retroperitoneal and bilateral common iliac and internal iliac lymph nodes. Bone marrow and lymph node biopsies were positive for AML. She was started on mitoxantrone, etoposide and cytarabine chemotherapy. Prior to transplant CSF and marrow were negative for disease but FDG-PET scan demonstrated persistently positive lymph nodes. She received TMLI 20 Gy (12 Gy to liver and brain), etoposide, and cyclophosphamide followed by matched related donor stem cell infusion. She remains relapse free at 6 years and 1 month with mild oral and cutaneous chronic GVHD.*

### TMLI integrated with reduced intensity conditioning fludarabine and melphalan in older patients (> 60 years old) with R/R acute leukemia

In patients older than 60 years of age, myeloablative regimens can lead to unacceptably high NRM rates of ~20% at 100 days and 40% at 3 years (35). In these patients reduced intensity conditioning (RIC) regimens have been used (36) and are better tolerated, but can be less cytotoxic and rely more on the graft-versus-leukemia (GVL) effects to eradicate disease. As a result RIC regimens can result in a significant increase in relapse rates and decrease in OS and relapse-free survival (RFS) (37). Attempts to add standard 9 Gy TBI to a RIC regimen to reduce relapse rates resulted in unacceptable toxicities (6). Organ sparing targeted radiotherapy such as TMLI is needed (11, 15, 38, 39), specifically for older patients with limited HCT options, who present frequently with more aggressive and chemo-resistant disease, are unable to tolerate standard myeloablative regimens (40, 41), and where reduced intensity conditioning (RIC) regimens can be associated with increased relapse rates (37).

Rosenthal et al. in a pilot trial (NCT 00544466) evaluated TMLI 12 Gy (1.5 Gy twice a day, days -7 to -4) added to an established RIC regimen of fludarabine (flu) (25 mg/m^2^/d Days -7 to -4) and melphalan (mel) (140 mg/m^2^ Day -2) in patients with R/R acute leukemia in patients > 60 years old or those who could not tolerate TBI containing regimens due to co-morbidities (11, 15). Flu-mel is a frequently used conditioning regimen in high risk acute leukemia patients in complete first or second remission (CR1/CR2), which is the reason TMLI was added for R/R acute leukemia with higher tumor burden. The target structures included bone/marrow, major lymph node regions and spleen (**Figure 2B**). Brain and testes were included in patients with ALL. Sixty-one patients were treated with a median age of 55 years (9-70 years) (15). Acute leukemia comprised 72% of the study population (AML 57% and ALL 17%). The most common toxicity was mucositis. All patients engrafted without delays. Median follow-up was 7.4 years. Five-year OS, event free survival (EFS), cumulative incidence of relapse (CIR), and NRM were 42%, 41%, 26%, and 33%, respectively. Results confirmed that the addition of 12 Gy TMLI to Flu/Mel was feasible, with favorable long-term outcomes and with a NRM rate that was comparable to flu/mel alone (42–45).

*Case example. A 65-year-old male with AML was entered on this trial with induction failure. He had failed to achieve remission after 7 + 3 induction chemotherapy, cladribine, cytarabine, idarubicin, decitabine, high dose cytarabine and NK cells, and anti-CD34 antibody-chemotherapy conjugate therapy. He was referred to City of Hope from another tertiary bone marrow transplant program for this trial. He received 12 Gy TMLI, fludarabine 25 mg/m^2^ for 5 days, melphalan 140 mg/m^2^ followed by matched related donor stem cell infusion. He remains relapse free at 5.5 years and has mild GVHD of the gut (mouth and stomach).*

A successor phase I trial (NCT 03490569) is ongoing and is focused on dose escalation in this same population. To reduce toxicities and allow for dose escalation, the regimen was modified to evaluate TMLI 12-20 Gy (1.5-2.0 Gy twice a day, days -9 to -5) combined with reduced doses of fludarabine (30 mg/m^2^/d Days -4 to -2) delivered after instead of concurrent with TMLI and reduced doses of melphalan (100 mg/m^2^ Day -2).

### TMLI added to strategies to reduce GVHD in patients with haplo-identical donors

Graft versus host disease (GVHD) remains a major cause of morbidity and mortality in patients undergoing allogeneic HCT (46). Strategies to reduce GVHD are especially important in patients without a matched donor such as those with haplo-identical donors. GVHD prophylactic regimens traditionally have used methotrexate, calcineurin or mTOR inhibitors, mycophenolate mofetil and anti-thymocyte globulins. Post-transplant cyclophosphamide (PTCy) is a potent immunosuppressive agent that has been successfully used in combination with a calcineurin or mTOR inhibitors to prevent GVHD in HLA-matched and haploidentical transplants in multiple studies (47–54). PTCy also can be used as a single-agent GVHD prophylaxis after myeloablative HLA-matched related or unrelated bone marrow transplant (47). The mechanisms of action pf PTCy are thought to involve preferential killing or functional impairment of alloreactive T cell and enrichment of the regulatory T cell population which are more resistant to PTCy (47, 55–58).

These strategies while reducing GVHD can reduce the graft versus leukemia (GVL) effects, potentially resulting in higher relapse rates, as demonstrated in patients with AML undergoing haplo-identical donor HCT with RIC conditioning regimens and PTCy (59). Al Malki et al. evaluated the addition of dose escalated TMLI to an established PTCy conditioning regimen (39) to further reduce relapse rates without contributing to NRM or GVHD. The target structures were bone and bone marrow, major lymph node regions and spleen. Liver and brain were treated to 12 Gy. In a phase I trial (NCT02446964) 31 patients [median age 37 (21–58)] with high-risk or R/R acute leukemias or myelodysplastic syndrome underwent haplo-identical HCT conditioning with TMLI, concurrent fludarabine (25 mg/m^2^/day on day -7 to day -3) and Cy (14 mg.kg/day on days -7 and -6). TMLI dose was escalated from 12 to 20 Gy. GVHD prophylaxis was PTCy (50 mg/kg/d on days +3 and +4) with tacrolimus/mycophenolate mofetil. All patients engrafted. With a follow-up of 23.9 months for the whole cohort, 2-year NRM was 13%; cumulative incidence of day 100 grade 2-4 and 3-4 acute GvHD were 52%, 6%, respectively and chronic GVHD at 2 years was 35%. For patients at the recommended phase 2 dose (RP2D) of 20 Gy (n=12) the 1-year relapse rate, PFS and OS were 17%, 74% and 83%; respectively. HaploHCT with TMLI with PTCy was safe and feasible, with promising low relapse rates achieved with acceptable GVHD or NRM.

A phase II trial in patients < age 60 with R/R acute leukemia is ongoing (NCT04262843). For patients age 60 or older with R/R acute leukemia a phase I haploidentical HCT trial (NCT 03490569) of TMLI 12-20 Gy (1.5-2.0 Gy twice a day, days -9 to -5), fludarabine (30 mg/m^2^/d Days -4 to -2), melphalan (100 mg/m^2^ Day -2) and PTCy (50 mg/m2 days +3 and +4) is ongoing.

### TMLI in patients with AML in complete remission undergoing allogeneic HCT

Allogeneic HCT is the preferred curative approach for patients with high risk AML in complete remission. Myeloablative TBI containing conditioning regimens are often utilized. Given the encouraging results in R/R disease, we extended the evaluation of TMLI to AML in CR1/CR2. Stein et al. evaluated in a pilot trial (NCT03467386) a novel conditioning regimen of TMLI 20 Gy without pretransplant chemotherapy, together with PTCy to reduce the risk of GVHD, in patients with AML in CR1/CR2 undergoing matched donor allogeneic HCT. The target structures were bone and bone marrow, major lymph node regions and spleen treated to 20 Gy and liver and brain treated to 12 Gy. Dose escalated TMLI was intended to reduce relapses and to offset the possible reduction in GVL from PTCy. In 18 patients [(median age 40 (19–56)] no grade 3-4 Bearman toxicities or toxicity-related deaths were observed. All patients engrafted without delays. The cumulative incidence of moderate-to-severe cGVHD was 11·9%. Disease relapse at 2 years was 16·7%. At a median follow up of 24.5 months, 2-year estimates of NRM, OS, relapse free survival, and GVHD-/relapse-free survival (GRFS) rate were 0%, 86·7%, 83·3%, and 59.3%, respectively (13). These results compare favorably to historical results at this center where GRFS was 39% at 2 years using TBI 13.2 Gy combined with Cy or etoposide and tacrolimus/sirolimus prophylaxis in patients with AML in remission (60). Based on these encouraging results a larger phase 2 trial has been initiated.

### Toxicities with TMI and TMLI

TMI and TMLI have the potential to reduce toxicities compared to TBI. Intermediate and long-term toxicity data were reported on 142 patients who received TMI or TMLI from 2005 to 2016 and who were entered on a study which prospectively followed patients up to 8 years after TMI or TMLI (61, 62). In addition to standard follow-up, thyroid panel, ophthalmologic exams, pulmonary function studies, serum creatinine, glomerular filtration rate, and urine analysis were performed at 100 days, 6 months, 1 year and annually up to 8 years. Median TMI dose was 14 Gy with most patients receiving either 12 Gy (n=64) or 16 Gy (n=30). Median follow-up (range) for all patients was 2 years (0–8) and for alive patients (n=50) 5.5 years (0–8). One patient developed reversible radiation pneumonitis (RP). The crude incidence of RP was 1/142 (0.7%). The cumulative incidence of infection and RP (I/RP) was 22.7% at 2 years post TMI. Mean lung dose (MLD) ≤ 8 Gy was associated with a significantly lower rate of I/RP (2-year CI 20.8% vs 31.8%, p=0.012). The incidence of hypothyroidism, cataract formation and radiation induced renal toxicity was 6.0%, 7.0% and 0%, respectively.

Toxicities appear to be less compared to that reported for conventional TBI. Renal toxicity can range between 0% and 46.7% (63, 64). Rates of hypothyroidism requiring medication replacement range from 10.5% to 12.0% (65–67) and cataract formation has been reported to be as high as 89-100% (63, 68). These toxicities have been shown to correlate with total dose and dose per fraction (8). Therefore, the lower rates of toxicity observed with TMI and TMLI are likely due to organ sparing and reduction in organ dose using IMRT compared to conventional opposed fields used to deliver TBI (69). With conventional TBI these organs would usually receive the full total body dose.

Lung doses in this study were lower than that reported with conventional TBI and probably explain the observed low incidence of radiation pneumonitis. This compares favorably to conventional TBI where radiation pneumonitis rates are approximately 28 to 31% even with the use of lung shielding and fractionation (70–72). Keeping MLD ≤ 8 Gy is recommended to limit the risk of pulmonary I/RP and is a dose constraint on all current trials. The lower incidence of intermediate to late toxicities and no cases of non-engraftment to date suggest that the higher dose rates with the current fractionation schedules do not contribute to organ or marrow dysfunction and are consistent with published pre-clinical studies (73, 74).

### Organ sparing on recurrence rates after TMLI

TMLI has raised concerns that organ sparing will spare leukemia cells and increase recurrence rates. We reported on extramedullary (EM) recurrences in the first 101 patients undergoing allogeneic HCT with TMLI at this center. With a median follow-up of 12.8 months, 13 patients developed EM relapses at 19 sites. There was no relationship between dose and EM relapse. Nine EM relapses occurred in full dose regions (≥ 12 Gy), 5 at sites receiving close to the full dose (10.1 to 11.4 Gy) and 5 at sites receiving 3.6 to 9.1 Gy (75). In multivariate analysis EM disease prior to HCT was the only predictor of EM relapse as has been reported by others (76–79). The risk of EM relapse was comparable to that seen with other HCT conditioning regimens (76, 80–82). These data suggest the use of TMLI does not increase the risk of relapse in non-target regions compared to other conditioning regimens. Our current practice is to continue to offer patients with a history of EM disease TMLI conditioning regimens on clinical trials. Sites of active EM disease sites prior to HCT are included in the target regions. EM relapse rates continue to be monitored on all TMLI trials at this center.

## Discussion and future directions

TBI remains a critically important component of the conditioning regimen in patients undergoing HCT with randomized trials continuing to demonstrate superior outcomes with TBI regimens compared with non-TBI regimens (83–88). The role of TBI is the elimination of malignant cells and to provide immunosuppression to prevent rejection of donor hematopoietic stem cells. Fractionation and organ shielding improve the therapeutic ratio of conventional TBI with reduced toxicities and improved outcomes (89–94). Increased toxicities associated with higher dose-rates diminishes above 5 cGy/minute and is further reduced if TBI is fractionated (73, 95–98).

There are limitations with conventional TBI. Dose escalation is not feasible due to increased toxicities and regimen related mortality. TBI is also not tolerated in older patients. TMI and TMLI were developed by our group to address these limitations and an unmet need, specifically patients with advanced, refractory or relapsed hematologic malignancies who have poor outcomes or cannot tolerate standard myeloablative conditioning regimens, and where attempts to improve outcomes by adding or dose escalating conventional total body irradiation (TBI) was not possible due to associated toxicities. The emphasis was on redefining and expanding the use of radiation containing conditioning regimens to a new group of patients.

Most patients treated on TMLI trials at this center are patients with R/R acute leukemia who have no standard-of-care HCT options. The results in this population are very encouraging when compared to historical results and demonstrate that: 1) TMLI results in lower incidences of toxicities compared to TBI (61); 2) all patients have successfully engrafted; 3) extramedullary relapses are infrequent and comparable to other conditioning regimens (75); 4) TMLI doses to 20 Gy, with etoposide and cyclophosphamide in younger patients (< 60 years old) is safe with a 1-year NRM rate of 6% and 2-year OS and PFS of 48% and 33%, respectively (12, 33); and 5) in older patients (≥ 60 years old), adding 12 Gy TMLI to fludarabine and melphalan is feasible with an NRM rate similar to fludarabine-melphalan alone, and with encouraging 5-year OS and EFS of 42% and 41%, respectively (15). The results of these studies warrant larger scale multi-center trials to confirm that these single center results can be reproduced.

More recently TMLI strategies at this center have been extended to other patient populations. Dose escalated TMLI has been added to a PTCy GVHD reduction regimen in R/R acute leukemia patients undergoing HCT with a haplo-identical donor with promising low relapse rates achieved without an increase in GVHD or NRM (39). This approach warrants further investigation given the initial promising results and rapid expansion of haplo-identical HCT.

Given the encouraging results in R/R disease, TMLI is now being evaluated in patients with AML in CR1 and CR2 undergoing matched donor allogenic HCT with results that compare favorably to similar patients treated with traditional TBI and conventional GVHD preventative regimens at this center. This approach is now being evaluated in a larger phase II trial which may eventually lead to a randomized trial evaluating this regimen as a possible alternative to current TBI or non-TBI containing conditioning regimens.

Since initiation of TMI trials at this center, other centers have reported their experience, although most of the experience remains limited to date. Detailed reviews of the TMI clinical experience have recently been published (38, 99–101). Most trials have evaluated myeloablative doses of TMI as part of the conditioning regimen prior to allogeneic HCT. A Phase I trial of TMI (3–12 Gy 1.5 Gy twice daily) with fludarabine (40 mg/m^2^/day × 4) and busulfan (4,800 μM∗min) reported a MTD of 9 Gy in patients 18-65 years of age with high risk disease. NRM was 29%, RFS was 43% and OS was 50% (102). A phase II trial of this regimen is ongoing. A Phase I trial combining dose-escalated TMI from 12 to 18 Gy (3 Gy/day) with fludarabine (25mg/m^2^ on days−9 to−7) and cyclophosphamide (60 mg/m^2^ on days −8 and −7), established an MTD of 15 Gy (103). Other groups are evaluating larger fraction sizes of up to 5 Gy (104). A phase I trial in patients with relapsed hematologic disease undergoing second or greater allogeneic HCT were treated with TMI doses of 6 to 12 Gy. The 2-year NRM, PFS and OS was 17%, 48% and 50%, respectively. The recommend TMI dose was 12 Gy in younger patients and 9 Gy in older patients (105). Initial results of an ongoing prospective pilot trial evaluating TMI and Cy in patients with high risk AML, ALL, or MDS who were older than 50 years old or with comorbidities unable to undergo TBI based regimens were reported (106). With a median follow-up of 14 months, relapse rate was 0% and median OS was 313 days (20–784).

In AML patients undergoing allogeneic HCT with a haplo-identical donor, a pilot trial reported results combining Treg/Tcon adoptive immunotherapy to reduce GVHD with myeloablative TBI or TMLI and low dose chemotherapy (107–109). The conditioning regimen included TBI for patients up to age 50 years and total marrow/lymphoid irradiation for patients age 51 to 65 years. TMLI was delivered in 2 daily fractions of 1.5 Gy (TMI) and 1.3 Gy (TLI) (total doses 13.5 Gy and 11.7 Gy respectively), followed by thiotepa, fludarabine, and cyclophosphamide. The probability of moderate/severe cGVHD/relapse-free survival was 75% (109).

Trials are in progress evaluating TMI instead of TBI in patients with acute leukemia in remission. At Beijing 307 Hospital a conditioning regimen of TMI 12 Gy in 4 Gy daily fractions combined with pre-transplant Cy (60 mg/kg/d x 2) and at the University Hospitals of Geneva TMI 12 Gy at 4 Gy per day with a simultaneous boost to active marrow to 13.5 Gy in older patients (40-80 years of age) are being evaluated. Other centers have combined TBI with TMI to select targets areas as a form of localized boost (110, 111).

TMI trials have also been reported in patients with multiple myeloma. A phase II trial reported results of 50 patients who underwent a tandem autologous HCT using TMI 12 Gy (4 Gy daily) conditioning followed by a second autologous HCT using melphalan 200 mg/m^2^. Additional boost to 24 Gy was delivered to active FDG-PET lesions. The 5-year OS and PFS were 74% and 55%, respectively (112). Other groups have administered TMI concomitantly with melphalan prior to autologous HCT. A phase I trial combined TMI with melphalan (200 mg/m^2^) in patients with relapsed or refractory disease (113). An MTD was not reached and the authors concluded that 9 Gy TMI could be combined safely with melphalan. A French multicenter Phase I TMI dose escalation trial evaluating TMI with melphalan (140 mg/m^2^) in first relapse patients has been completed (114). TMI as a single modality (14, 16 and 20 Gy at 2 Gy twice daily) in 9 patients with relapsed multiple myeloma prior to autologous HCT has been reported (115). No dose limiting toxicities were observed. A phase I trial at this center is evaluating the combination of 9 Gy TMI with the RIC regimen of fludarabine and melphalan in advanced patients undergoing allogeneic HCT (116).

The implementation of TMI and TMLI has led to using IMRT to deliver TBI (IM-TBI) at this center (NCT04281199) and other centers primarily in patients with acute leukemia in complete remission undergoing allogeneic HCT (117, 118). At this center IM-TBI is performed on prospective clinical trials with uniformly applied dose constraints to reduce bias and to have a IM-TBI clinical guideline foundation upon which to build on in future clinical trials. Our main reasons for implementing IM-TBI are to reduce lung dose and to accurately track radiation dose in TBI patients. Conventional 2D TBI dosimetry only provides an estimate of organ and total body dose as it does not use 3D CT based dosimetry. Both helical tomographic and VMAT IMRT can be used to deliver IM-TBI (119–123). Advantages include improved dose uniformity and improved sparing of critical organs compared to conventional TBI delivery methods (119). This has the potential to reduce toxicities, as predicted from studies of patients undergoing TBI (124) or TMI (61) which demonstrate that a mean lung dose of ≤ 8 Gy results in an increase in OS or a decrease in pulmonary toxicity, respectively. It is important to note that IM-TBI and TMI/TMLI are non-competing approaches being evaluated in different patient groups. Also, unlike TMI or TMLI, myeloablative doses of IM-TBI in older patients and dose escalation is not feasible due to increased toxicities. This will only be possible with dose distributions that spare more normal organs and approach that of TMI and TMLI. As a result, IM-TBI is limited to the same HCT populations that undergo conventional TBI and will have to demonstrate reduced toxicities and superior outcomes if it is to replace conventional TBI.

Combining different forms of targeted systemic radiotherapy such as TMLI and targeted radiopharmaceutical therapy should be evaluated. TMI/TMLI can be viewed as a form of targeted “systemic” radiotherapy that utilizes CT imaging to target anatomic regions likely to harbor disease. Another form of targeted systemic radiotherapy is radioimmunotherapy (RIT) which utilizes radiolabeled monoclonal antibodies to target cancer cells that express a specific antigen. Antibodies directed against CD45, CD33, CD66, CD22, CD25, and CD20 radiolabeled with ^131^I, β-emitters, or α-emitters have been evaluated in clinical trials as single agents, in combination with other therapies, incorporated in HCT conditioning regimens as an alternative to TBI (125–137), or combined with myeloablative doses of TBI (138–141) in patients with hematologic malignancies. A phase I trial at this center is currently evaluating the combination of RIT added to 12 Gy TMLI, fludarabine and melphalan in R/R acute leukemia undergoing matched donor allogenic HCT (NCT05204147). TMLI and RIT are potentially complementary and can address the limitations of each modality. The addition of RIT to TMLI can add additional dose to cancer cells, including cancer cells in circulation and in organs not targeted by TMLI. With RIT there is unintended normal organ uptake, making TMLI better positioned to be combined with RIT than standard TBI. Finally, radiolabeled antibodies and other radiopharmaceuticals are being evaluated as molecular imaging agents at this center to refine targeting of TMLI in the future (142, 143).

## Conclusion

**Figure 3:**
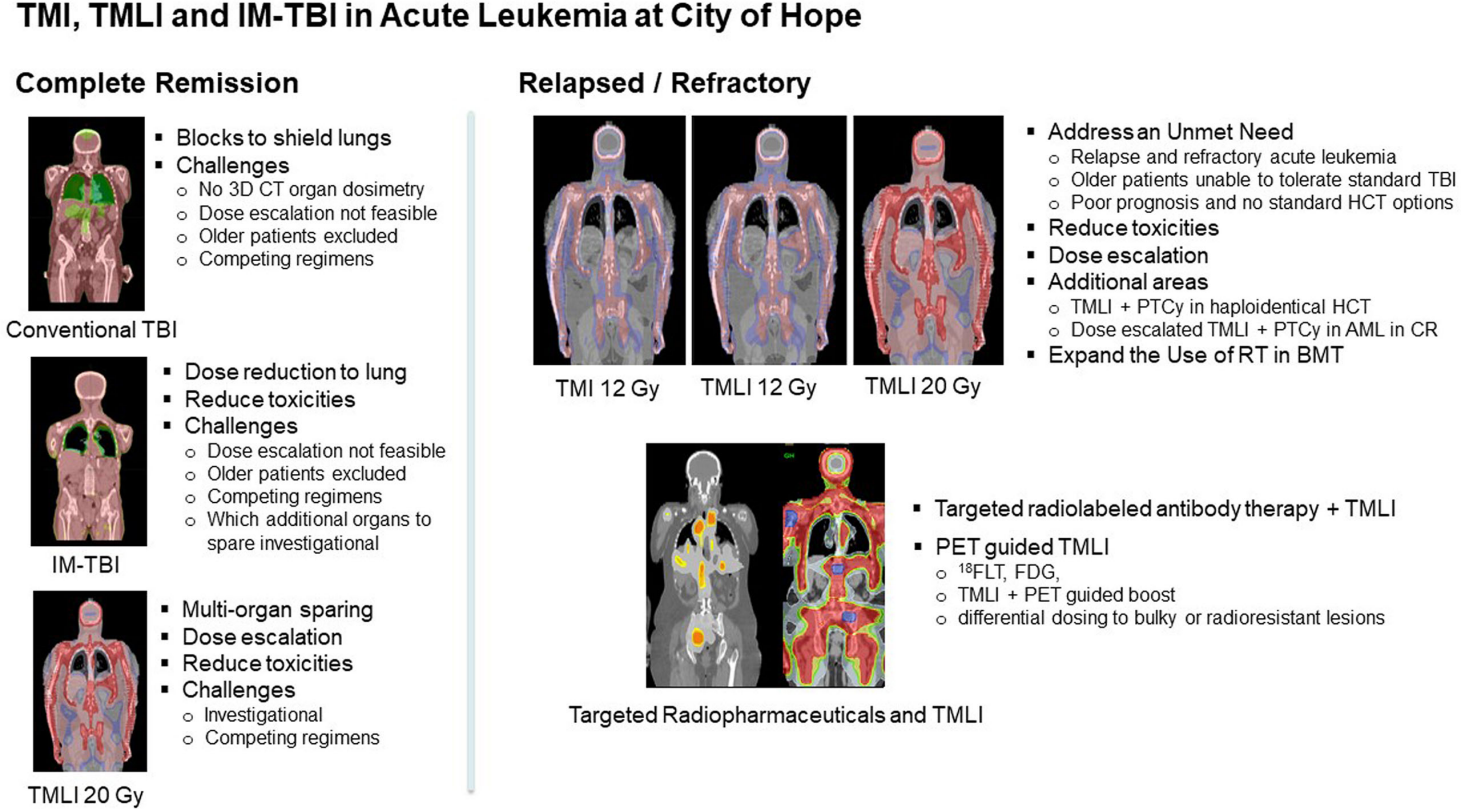
Overview of TMI, TMLI and IM-TBI clinical applications in patients with acute leukemia undergoing allogeneic hematopoietic cell transplantation at this center.

TMI and TMLI continue to be actively investigated at this center and at an increasing number of centers worldwide which has been recently reviewed (144, 145). **Figure 3** summarizes the clinical trial strategies currently ongoing at this center. Clinical results show significant promise and demonstrate the ability to offer TMI and TMLI to older patients or those with co-morbidities, regimen related toxicity and mortality rates that are in general lower compared to standard myeloablative conditioning regimens, the ability to dose escalate, and encouraging response and survival rates in R/R disease. The initial concerns of increased toxicities, engraftment failure and relapses due to higher dose-rates and organ sparing have not been observed and prospective trials continue to monitor this. These results support the use of helical tomographic or volumetric arc based IMRT for the delivery of TMI and TMLI in future clinical trials.

This rapidly developing field should move towards developing uniform planning and treatment guidelines and standardized reporting of organ doses and dose-rates. Multicenter trials are needed to confirm results reported by single institutions and to answer important questions that remain. The optimum fractionation schedules, fraction sizes, chemotherapy agents, and chemotherapy/TMI/TMLI sequencing need to be defined. Future clinical trials will investigate radiation dose distributions which will span a continuous spectrum ranging from IM-TBI with lung sparing, to IM-TBI with multi-organ sparing, to TMI/TMLI without dose escalation, and to TMI/TMLI with dose escalation. The appropriate dose distributions, target regions, target doses, and organs to spare for each clinical scenario need to be determined. Ultimately clinical trials need to demonstrate that TMI based conditioning regimens offer advantages over current approaches.

In conclusion, the development and implementation of TMI and TMLI requires a collaborative effort between radiation oncology and hematology to apply technology advances in radiation oncology to address an unmet need in HCT and to shift the paradigm towards more effective and safer strategies to integrate radiation therapy into HCT conditioning beyond what is possible with TBI. TMI and TMLI hold significant promise in redefining and expanding the role of radiotherapy in hematopoietic cell transplantation.

## Author contributions

JW performed the summary of clinical results and manuscript preparation. AL contributed to the methodology summary and images used in all figures. CH contributed to the methodology summary and reporting of organ dose estimates. All authors (JW, AL, CH, SD, TS, JP, DY, GS, ASa, SH, MA, JR and ASt) provided input and critical review of the manuscript and were actively involved as investigators of the clinical trials and studies reviewed in this manuscript. ASt, JR, MA, GS, SD, SH and JW were principal investigators of the prospective trials and prospective studies summarized in this review. All authors contributed to the article and approved the submitted version.

## Conflict of interest

The authors declare that the research was conducted in the absence of any commercial or financial relationships that could be construed as a potential conflict of interest.

## Publisher’s note

All claims expressed in this article are solely those of the authors and do not necessarily represent those of their affiliated organizations, or those of the publisher, the editors and the reviewers. Any product that may be evaluated in this article, or claim that may be made by its manufacturer, is not guaranteed or endorsed by the publisher.
